# Supplementary material for: Atomic representation and algorithms for polytomous knowledge spaces
Source: PLoS One. 2026 Apr 17;21(4):e0346061. doi: 10.1371/journal.pone.0346061 (PMC13089903; doi:10.1371/journal.pone.0346061)
Supplement: Attachment — Submitted filename: Review Report.pdf [file pone.0346061.s001.pdf]

Review Report for Manuscript PONE-D-25-55989

Overall Assessment:

This manuscript presents a well-structured and theoretically sound contribution to the field of knowledge space theory (KST). It addresses a significant limitation of classical KSTthe dichotomous response modelby introducing a novel framework for polytomous knowledge structures based on closure spaces and atomic decomposition. The work is mathematically rigorous, clearly presented, and offers practical algorithms. It is suitable for publication pending minor revisions to improve clarity and correct some English language issues.

Major Strengths:

1. Novel Contribution: The introduction of polytomous closure spaces and the associated atomic decomposition theory provides a fresh and computationally promising approach to handling graded knowledge.

2. Theoretical Rigor: The lattice-theoretic foundation is solid, and the proofs are clearly articulated. The duality between knowledge spaces and closure spaces is elegantly extended.

3. Practical Relevance: The development of efficient algorithms (base extraction and space generation) that leverage the atomic structure is a key strength, bridging theory and application.

4. Clarity of Exposition: The paper is generally well-organized, with helpful examples (e.g., the diamond lattice) that illustrate complex concepts.

Suggestions for Improvement and Revisions:

1. Comparison of Polytomous Knowledge Structures:

• The concept of polytomous knowledge structure is an important concept in polytomous KST. Note that there are some different descriptions for polytomous knowledge structure. Please give some Comparisons among Definition 2, Defini-

tion 1 in [Journal of Mathematical Psychology 110 (2022) 102708] and Definition 1 in [Journal of Mathematical Psychology 113 (2023) 102743].

2. Enhanced Algorithm Discussion:

• The complexity analyses for Algorithms 1-4 are helpful but could be strengthened. For Algorithm 2, please explicitly state its worst-case time complexity as $O(N^2 \cdot |Q|)$ in the main text, not just in the analysis block.

• Consider adding a brief comparative discussion or a small table summarizing the time/space complexity and preferred use cases for Algorithms 1 vs. 2 and Algorithms 3 vs. 4. This would help readers choose the appropriate method.

3. Clarification of Scope and Limitations:

• The Granularity condition (Definition 13) is crucial for the complete atomic decomposition. Please add a sentence or two in Section 3.2 discussing what types of knowledge structures might not be granular and the implications of this limitation for the proposed framework's applicability.

• In the conclusion, the future work direction about "incremental update methods for dynamic knowledge structures" is excellent. Please briefly hint at why this is challenging within the current atomic framework.

4. Conclusion Strengthening:

• The conclusion effectively summarizes the contributions. To make it more impactful, consider adding a single sentence that more explicitly states the practical advantage over the reductionist approach (e.g., "This direct representation avoids the combinatorial explosion inherent in the virtual-item reductionist method, enabling scalable assessment for realistic item banks.").

Corrections for English Language and Typographical Errors:

• Page 4, Remark 1: "This discrete formulation facilitates efficient algorithmic implementation and combinatorial analysisadvantages that are crucial..." C Insert a space: "analysis advantages". Also, "assessmentwhile".

• Page 5, Definition 5: The formatting "(i) Extensivity:" appears on a line by

itself. It is preferable to keep the axiom label and its description on the same line for readability.

• Page 6, after Definition 11: “Example 1 represents an extreme case...” This paragraph reads as a continuation of the definition text. Consider adding a header like “Discussion” or starting a new paragraph to separate the example interpretation from the formal definition.

• Page 11, Figure Caption: “Figure 1: Diamond-shaped value lattice V_p for each item $p \in Q$”– Ensure this figure is correctly placed and referenced in the final manuscript.

• Page 12, Algorithm 1 Pseudo-code: The line “Complexity Analysis of Algorithm 4.1:” seems to be a formatting error. It should likely be a separate, plain-text section header.

• Page 14, before Example 5: The sentence “The direct approach, while correct, has cubic complexity in worst case.” is incomplete. Suggest: ”The direct approach (Algorithm 1), while correct, has cubic time complexity in the worst case.”

• Page 18, Theorem 4 Proof, (b) $\Rightarrow$ (a) direction: The final parenthetical statement “hence $M \sqcup B = M$” is cut off awkwardly. Consider revising to: “... hence $M \sqcup B = M$”.

• Page 20 (Text Layer): The formatting is inconsistent, mixing bullet points and dashes. In the final version, this should be formatted as proper pseudo-code or a consistent descriptive list.

• Throughout: The word “polytomous” is sometimes misspelled as “polydomous” in the text body (e.g., Page 22, “polydomous knowledge structures”). Please ensure consistent use of “polytomous” as in the title and abstract.

• Minor Punctuation: Check for consistent use of the Oxford comma and spacing around mathematical operators.

Recommendation:

Accept with Minor Revisions. The manuscript presents significant theoretical and practical contributions to polytomous KST. Addressing the points above will further enhance its clarity, rigor, and readiness for publication.
