# Supplementary material for: Atomic representation and algorithms for polytomous knowledge spaces
Source: PLoS One. 2026 Apr 17;21(4):e0346061. doi: 10.1371/journal.pone.0346061 (PMC13089903; doi:10.1371/journal.pone.0346061)
Supplement: Attachment — Submitted filename: Review-report-on-PONE-D-25-55989 .pdf [file pone.0346061.s002.pdf]

Manuscript: PONE-D-25-55989
Title: Efficient Atomic Representation for Polytomous Knowledge Structures
Recommendation: Minor Revision

Overall Impression

The manuscript presents a well-structured and theoretically sound framework for polytomous knowledge structures based on atomic decomposition. The work addresses an important gap in the literature and offers valuable theoretical insights and practical algorithms. The writing is clear and the contributions are substantial. I recommend acceptance after addressing the following relatively minor revisions.

Specific Revisions Requested

1. **Page 2, Paragraph 2:** Please revise the sentence to: "The relationship between skills and **items** has been formalized through skill maps and competence-performance approaches."

2. **Page 2, Paragraph 3:** Please correct the statement to: "which establishes a one-to-one correspondence between quasi-orders on the item set and **quasi ordinal spaces**."

3. **Page 4:** Typographical errors:
   - "itemessential" → "item essential"
   - In Remark 1: "analysisadvantages" → "analysis advantages"; "assessmentwhile" → "assessment while"

4. **Page 4, Definition 4:** Please add an appropriate citation, e.g., Stefanutti (2020).

5. **Page 5-6, Definition 10:** Please clarify the notation: either write $\mathcal{B}(\mathcal{K}) = J_{\text{irr}}(\mathcal{K})$ or explicitly define $J_{\text{irr}}(\mathcal{K})$ as a set.

6. **Page 6, Definition 9:** Please define $J_{\text{irr}}(V_p)$ (join-irreducible elements of V_p) before use.

7. **Page 6, Theorem 1 Proof (Sufficiency):** Please justify why for each join-irreducible $w \leq_q K(q)$, there exists $J_{q,w} \in \mathcal{K}$ with $J_{q,w} \sqsubset K$.

8. **Page 8, Proposition 2:** Please state explicitly that each $(V_p, \leq_p)$ is assumed to be a Boolean algebra for De Morgan's Law to apply.

9. **Page 11, Example 3:** Please include the referenced "Figure 1: Diamond-shaped value lattice" and clarify that in this lattice, 3 is not an atom (since $3 = 1 \vee 2$).

10. **Page 12-13, Algorithm 1:** Please state the time complexity explicitly, e.g., $O(N^3 \cdot |Q|)$ where $N = |\mathcal{K}|$.

11. **Page 14, Algorithm 2:** For consistency, please provide formal pseudocode similar to Algorithms 1, 3, and 4.

12. **Page 19, Algorithm 4:** Please define variables t and m in the complexity analysis (e.g., $t = |\mathcal{B}|$, $m = |Q|$).

13. **Throughout the manuscript:** Please standardize variable usage for items (consistently use $p \in Q$).

14. **References:** Please adopt a consistent citation format (currently mixed numbered and author-year).
