# Supplementary material for: Atomic representation and algorithms for polytomous knowledge spaces
Source: PLoS One. 2026 Apr 17;21(4):e0346061. doi: 10.1371/journal.pone.0346061 (PMC13089903; doi:10.1371/journal.pone.0346061)
Supplement: Attachment — Submitted filename: Response-to-Reviewers.pdf [file pone.0346061.s003.pdf]

To: Dr. Fucai Lin, Academic Editor, PLOS ONE
Manuscript ID: PONE-D-25-55989
Title: Atomic representation and algorithms for polytomous knowledge spaces

Dear Dr. Lin and Reviewers,

Thank you for the opportunity to revise my manuscript titled "Atomic Representation and Algorithms for Polytomous Knowledge Spaces" (Revised from "Efficient Atomic Representation for Polytomous Knowledge Structures", Manuscript ID: PONE-D-25-55989). I sincerely appreciate the reviewers' thorough and constructive comments, which have guided us in substantially improving the paper.

In response to the reviewers' feedback, I have made detailed revisions as outlined in the point-by-point responses below. Additionally, I have conducted a comprehensive review of the manuscript and implemented further improvements to enhance its clarity, rigor, and presentation. All changes are clearly marked in the updated manuscript for your convenience.

Below I provide a point-by-point response to each reviewer's comments.

RESPONSE TO REVIEWERS' COMMENTS

Reviewer 1

Overall Assessment: This manuscript presents a well-structured and theoretically sound contribution to the field of knowledge space theory (KST). It addresses a significant limitation of classical KST—the dichotomous response model—by introducing a novel framework for polytomous knowledge structures based on closure spaces and atomic decomposition. The work is mathematically rigorous, clearly presented, and offers practical algorithms. It is suitable for publication pending minor revisions to improve clarity and correct some English language issues.

Major Strengths:

1. Novel Contribution: The introduction of polytomous closure spaces and the associated atomic decomposition theory provides a fresh and computationally promising approach to handling graded knowledge.
2. Theoretical Rigor: The lattice-theoretic foundation is solid, and the proofs are clearly articulated. The duality between knowledge spaces and closure spaces is elegantly extended.
3. Practical Relevance: The development of efficient algorithms (base extraction and space generation) that leverage the atomic structure is a key strength, bridging theory and application.
4. Clarity of Exposition: The paper is generally well-organized, with helpful examples (e.g., the diamond lattice) that illustrate complex concepts.

I sincerely thank the reviewer for the positive assessment and constructive comments. The specific points raised by the reviewer are addressed in detail below. All page and section numbers refer to the revised manuscript (PONE-D-25-55989.pdf).

Suggestions for Improvement and Revisions

1. **Comparison of polytomous knowledge structures:**
   - The concept of polytomous knowledge structure is an important concept in polytomous KST. Note that there are some different descriptions for polytomous knowledge structure. Please give some comparisons among Definition 2, Definition 1 in [Journal of Mathematical Psychology 110 (2022) 102708] and Definition 1 in [Journal of Mathematical Psychology 113 (2023) 102743].

   Response: I am grateful to the reviewer for this valuable suggestion. To provide the requested clarification, I have added a comprehensive comparative discussion in Section 3.1 (immediately after Definition 3.1) as Remark 3.1 (pp. 5-6). This remark systematically contrasts our definition with those in the literature, addressing:

   (i) the coverage condition in its various formulations (function vs. transversal views);

(ii) the differences between our definition and Heller's [22] (bounded poset vs. lattice; infinite vs. finite item sets);

(iii) the relationship with Stefanutti et al.'s framework [33] and Ge's works [18,19] highlighting the distinctions between uniform and item-specific scales, as well as the treatment of the trivial states $K_\perp$ and $K_\top$.

The remark concludes by noting that our definition accommodates items with different rating formats (e.g., 3-point vs. 5-point scales), whereas Stefanutti et al. and Ge require a uniform scale.

2. **Enhanced algorithm discussion:**

   - The complexity analyses for Algorithms 1–4 are helpful but could be strengthened. For Algorithm 2, please explicitly state its worst-case time complexity as $O(N^2 \cdot |Q|)$ in the main text, not just in the analysis block.
   - Consider adding a brief comparative discussion or a small table summarizing the time/space complexity and preferred use cases for Algorithms 1 vs. 2 and Algorithms 3 vs. 4. This would help readers choose the appropriate method.

   Response: I appreciate this suggestion for improving algorithmic clarity. I have implemented both enhancements:

   (1) Explicit Complexity Statement: In Section 4.1 (p. 16 of the revised manuscript), I have added an explicit statement of Algorithm 2's worst-case time complexity in the Complexity Analysis block, where it now reads: "Achieves $O(N^2 \cdot m)$ time, which is optimal for pairwise-comparison-based approaches." (Here $m = |Q|$ denotes the number of items.)

   (2) Comparative Analysis Table: I have added a new Subsection 4.3 titled "Algorithm Comparison and Selection" (p. 21), which features Table 1. This table systematically compares all four algorithms, summarizing their time complexity, space complexity, and preferred use cases. This enables readers to make informed decisions based on their specific problem scales and requirements.

   These enhancements add a complexity statement and a comparison table to the algorithm section.

3. **Clarification of scope and limitations:**

   - The Granularity condition (Definition 13) is crucial for the complete atomic decomposition. Please add a sentence or two in Section 3.2 discussing what types of knowledge structures might not be granular and the implications of this limitation for the proposed framework's applicability.
   - In the conclusion, the future work direction about 'incremental update methods for dynamic knowledge structures' is excellent. Please briefly hint at why this is challenging within the current atomic framework.

   Response: I have enriched these discussions as suggested, adding important context about both current limitations and future challenges:

   (1) Scope of Granularity: In Section 3.2 (p. 11, Remark 3.5), I now explicitly discuss non-granular structures. I use the diamond lattice as a concrete example where the top element $3 = 1 \vee 2$ is not join-irreducible, explaining that granularity requires $V_p \setminus \{\perp_p\} = J_{\mathrm{irr}}(V_p)$. This clarification helps readers understand the trade-off: while limiting admissible value lattices, this constraint ensures complete atomic decomposition, which is essential for our representation framework.

   (2) Future Work Challenges: In the Conclusion (Section 5, p. 21), I have expanded the discussion on incremental updates to explicitly note the technical challenges. I explain that when new items are added or prerequisites change, the atomic base $\mathcal{A}(\mathcal{K})$ may require non-local recomputation, potentially breaking the bijective correspondence between $\mathcal{K}$ and $\mathcal{A}(\mathcal{K})$. This transparency about limitations provides valuable direction for future research while contextualizing our current contributions.

4. **Conclusion strengthening:**

   - The conclusion effectively summarizes the contributions. To make it more impactful, consider adding a single sentence that more explicitly states the practical advantage over the reductionist approach (e.g., 'This direct representation avoids the combinatorial explosion inherent in the virtual-item reductionist method, enabling scalable assessment for realistic item banks.').

Response: I have incorporated the reviewer's excellent suggestion verbatim to strengthen the conclusion's impact. The added sentence now appears in Section 5 (page 21):

> This direct representation avoids the combinatorial explosion inherent in the virtual-item reductionist method, making scalable assessment feasible for realistic item banks with polytomous response options.

This addition clearly articulates the practical advantage of our approach and makes the conclusion more memorable and impactful.

Corrections for English Language and Typographical Errors

1. **Page 4, Remark 1:** "This discrete formulation facilitates efficient algorithmic implementation and combinatorial analysisadvantages that are crucial..." C Insert a space: "analysis advantages". Also, "assessmentwhile".

   Response: I thank the reviewer for pointing out these typographical errors. Upon revision, I have restructured Section 2, and the original Remark 1 has been removed as part of this reorganization. The text containing these terms no longer appears in the manuscript.

2. **Page 5, Definition 5:** The formatting "(i) Extensivity:" appears on a line by itself. It is preferable to keep the axiom label and its description on the same line for readability.

   Response: Reformatted as suggested. In Definition 2.4 (p. 3), each axiom now appears with its label and condition on a single line, e.g., "(i) $x \leq \phi(x)$". The axiom names (extensivity, monotonicity, idempotence) have been removed to streamline the presentation, as they were not essential for the definition.

3. **Page 6, after Definition 11:** "Example 1 represents an extreme case..." This paragraph reads as a continuation of the definition text. Consider adding a header like "Discussion" or starting a new paragraph to separate the example interpretation from the formal definition.

   Response: I thank the reviewer for this suggestion. In the revised manuscript, I have restructured Section 3. All examples (now Examples 3.2–3.4 on pp. 8-9) are presented as separate paragraphs with clear headings. The interpretative discussion that originally followed each example has been consolidated into Remark 3.5 (pp. 9-10), which provides a unified analysis of the examples' significance and limitations.

4. **Page 11, Figure Caption:** "Figure 1: Diamond-shaped value lattice V_p for each item $p \in Q$" – Ensure this figure is correctly placed and referenced in the final manuscript.

   Response: Corrected as suggested. In Example 3.6 (p. 13 of the revised manuscript), Figure 1 now appears with the correct caption: "Figure 1: Diamond-shaped value lattice V_p for each item $p \in Q$". The figure is properly placed and referenced in the text.

5. **Page 12, Algorithm 1 Pseudo-code:** The line "Complexity Analysis of Algorithm 4.1:" seems to be a formatting error. It should likely be a separate, plain-text section header.

   Response: I thank the reviewer for pointing out this formatting issue. In the revised manuscript (pp. 14-15), the complexity analysis now appears as a separate plain-text section header "Complexity Analysis of Algorithm 1", clearly distinguished from the pseudo-code. The analysis itself has also been rewritten as a coherent paragraph for improved readability.

6. **Page 14, before Example 5:** The sentence "The direct approach, while correct, has cubic complexity in worst case." is incomplete. Suggest: "The direct approach (Algorithm 1), while correct, has cubic time complexity in the worst case."

   Response: I thank the reviewer for this suggestion. In the revised manuscript (p. 15), the discussion has been expanded to provide a more complete comparison. The text now explicitly states that Algorithm 1 has exponential worst-case time complexity $O(N \cdot 2^N \cdot |Q|)$ and recommends Algorithm 2 with quadratic complexity $O(N^2 \cdot |Q|)$ for practical applications.

7. **Page 18, Theorem 4 Proof, (b) $\Rightarrow$ (a) direction:** The final parenthetical statement 'hence $M \sqcup B = M$' is cut off awkwardly. Consider revising.

   Response: Revised as suggested. In the proof of Theorem 4 (p. 18 of the revised manuscript), the (b) $\Rightarrow$ (a) direction now ends with a complete sentence: "Therefore, all base elements contained in K are also contained in M, which means $K \sqsubseteq M$." The awkwardly cut-off parenthetical has been removed.

8. **Page 20 (Text Layer):** The formatting is inconsistent, mixing bullet points and dashes. In the final version, this should be formatted as proper pseudo-code or a consistent descriptive list.

   Response: Reformatted as a consistent descriptive list. The content of the original Example 7 now appears as Example 4.4 (pp. 19-20) accompanying Algorithm 4, with the formatting corrected as suggested.

9. **Throughout:** The word “polytomous” is sometimes misspelled as “polydomous” in the text body (e.g., Page 22, “polydomous knowledge structures”). Please ensure consistent use of “polytomous’.

   Response: I have corrected all instances of “polydomous’ to “polytomous’ throughout the manuscript.

10. Minor Punctuation: Check for consistent use of the Oxford comma and spacing around mathematical operators.

    Response: I have conducted a comprehensive review of the manuscript to ensure consistent punctuation (including Oxford comma usage) and proper spacing around mathematical operators throughout.

Recommendation:

Accept with Minor Revisions. The manuscript presents significant theoretical and practical contributions to polytomous KST. Addressing the points above will further enhance its clarity, rigor, and readiness for publication.

Response: I am grateful for the positive recommendation and thank the reviewer for their meticulous suggestions, which have significantly improved the precision and readability of our manuscript. All suggested corrections have been implemented in the revised version. In addition to the changes requested by the reviewer, we have also made further enhancements to strengthen the manuscript, including substantial restructuring of Sections 2 and 3, a revised title, refined abstract and keywords, and an updated author affiliation. These improvements are detailed in the cover letter.

Reviewer 2

Overall Impression

The manuscript presents a well-structured and theoretically sound framework for polytomous knowledge structures based on atomic decomposition. The work addresses an important gap in the literature and offers valuable theoretical insights and practical algorithms. The writing is clear and the contributions are substantial. I recommend acceptance after addressing the following relatively minor revisions.

Response: I sincerely thank the reviewer for the detailed and constructive feedback, which has helped us improve the manuscript’s precision, clarity, and consistency. All requested revisions have been carefully implemented. Below I provide a point-by-point response; all page numbers refer to the revised manuscript (PONE-D-25-55989.pdf).

Specific Revisions Requested

1. **Page 2, Paragraph 2:** Please revise the sentence to: "The relationship between skills and **items** has been formalized through skill maps and competence-performance approaches."

   Response: I have revised the sentence as suggested. In Section 1 (p. 2) of the revised manuscript, the sentence now reads: "The relationship between skills and items has been formalized through skill maps [11, 12] and competence-performance approaches [1, 26]."

2. **Page 2, Paragraph 3:** Please correct the statement to: "which establishes a one-to-one correspondence between quasi-orders on the item set and quasi ordinal spaces."

   Response: I have corrected the statement. In Section 1 (p. 2), the text now states: "which establishes a one-to-one correspondence between quasi-orders on the item set and quasi ordinal spaces [2, 10]."

3. **Page 4:** Typographical errors:

   - "itemessential" $\rightarrow$ "item essential"
   - In Remark 1: "analysisadvantages" $\rightarrow$ "analysis advantages"; "assessmentwhile" $\rightarrow$ "assessment while"

   Response: I thank the reviewer for pointing out these typographical errors. Upon revision, the content on page 4 has been substantially restructured. The text containing "itemessential" no longer appears in the manuscript. Similarly, Remark 1 has been completely rewritten and expanded as Remark 3.1 (pages

5-6); the sentence containing "analysisadvantages" and "assessmentwhile" has been removed during this revision. As a result, these typographical errors have been addressed through content reorganization rather than isolated corrections.

4. **Page 4, Definition 4:** Please add an appropriate citation, e.g., Stefanutti (2020).

   Response: I have addressed this suggestion by adding a comparative discussion immediately following the definition. In the revised manuscript, the definition of a polytomous knowledge space is Definition 3.2 (p. 6). I note that while Stefanutti et al. [33] work in a different framework (requiring a common complete lattice for all items), our definition aligns more closely with Heller's approach [22] while generalizing it to bounded posets and allowing infinite item sets.

5. **Page 5-6, Definition 10:** Please clarify the notation: either write $\mathcal{B}(\mathcal{K}) = J_{\text{irr}}(\mathcal{K})$ or explicitly define $J_{\text{irr}}(\mathcal{K})$ as a set.

   Response: I have clarified the notation. In Definition 3.6 (p. 6), I explicitly define: "The set $\mathcal{B}(\mathcal{K}) = J_{\text{irr}}(\mathcal{K})$ is called a base of $\mathcal{K}$."

6. **Page 6, Definition 9:** Please define $J_{\text{irr}}(V_p)$ (join-irreducible elements of V_p) before use.

   Response: I have added the definition. In Definition 2.5 (pp. 3-4), I now include: "The set of all completely join-irreducible elements of L is denoted by $J_{\text{irr}}(L)$." This definition precedes its first use in Definition 3.4 (p. 7).

7. **Page 6, Theorem 1 Proof (Sufficiency):** Please justify why for each join-irreducible $w \leq_q K(q)$, there exists $J_{q,w} \in \mathcal{K}$ with $J_{q,w} \sqsubset K$.

   Response: I have added the necessary justification. In Theorem 3.1 (p. 7), the ($\supseteq$) direction of the proof now explicitly states:

   > "This implies that for each such (q, w), there exists a state $J_{q,w} \in \mathcal{K}$ satisfying:
   > (i) $J_{q,w}(q) = w$;
   > (ii) $J_{q,w} \sqsubseteq K$;
   > (iii) $J_{q,w} \neq K$ (otherwise K would be minimal for (q, w))."

   This justification follows from the coverage condition (Definition 3.1) and the definition of atoms.

8. **Page 8, Proposition 2:** Please state explicitly that each $(V_p, \leq_p)$ is assumed to be a Boolean algebra for De Morgan's Law to apply.

   Response: I have clarified this assumption. In Proposition 3.2 (p.10), I now begin: "Let $(\mathcal{K}, \sqsubseteq)$ be a polytomous knowledge space where each $(V_p, \leq_p)$ is a Boolean algebra."

9. **Page 11, Example 3:** Please include the referenced "Figure 1: Diamond-shaped value lattice" and clarify that in this lattice, 3 is not an atom (since $3 = 1 \vee 2$).

   Response: I have included the figure and added clarification. Figure 1 (p. 13) now shows the diamond-shaped lattice, and in Example 3.6 (p.13), I explicitly note: "Note that the diamond lattice *cannot appear in any granular polytomous knowledge space* because its top element $3 = 1 \vee 2$ is not join-irreducible, violating the condition $V_p \setminus \{\perp_p\} = J_{\text{irr}}(V_p)$ required by granularity."

10. **Page 12-13, Algorithm 1:** Please state the time complexity explicitly, e.g., $O(N^3 \cdot |Q|)$ where $N = |\mathcal{K}|$.

    Response: I thank the reviewer for this suggestion. In the revised manuscript (pp. 14-15), I have provided an explicit complexity analysis for Algorithm 1, stating that its worst-case time complexity is $O(N \cdot 2^N \cdot |Q|)$. While this is higher than the suggested $O(N^3 \cdot |Q|)$, it accurately reflects the exponential nature of the algorithm due to its enumeration of all subsets of lower states. This explicit statement helps readers understand the algorithm's computational limitations.

11. **Page 14, Algorithm 2:** For consistency, please provide formal pseudocode similar to Algorithms 1, 3, and 4.

    Response: I have provided formal pseudocode for Algorithm 2. On p. 15, Algorithm 2 is now presented with the same formal structure as the other algorithms, including clear input/output specifications and step-by-step instructions.

12. **Page 19, Algorithm 4:** Please define variables t and m in the complexity analysis (e.g., $t = |\mathcal{B}|$, $m = |Q|$).

    Response: I have defined all variables. In the complexity analysis of Algorithm 4 (p. 19), I now explicitly state:

    > "Let $t = |\mathcal{A}|$ denote the number of atoms (base size), $m = |Q|$ the number of items, and $N = |\mathcal{K}|$ the number of generated states."

13. **Throughout the manuscript:** Please standardize variable usage for items (consistently use $p \in Q$).

    Response: I have standardized variable usage throughout the manuscript. All references to items now consistently use $p \in Q$ in definitions, examples, and main text. In proof contexts where multiple item indices are needed, I occasionally use q as a secondary variable (e.g., in Theorem 3.1), but the primary notation throughout is $p \in Q$.

14. **References:** Please adopt a consistent citation format (currently mixed numbered and author-year).

    Response: I have standardized the citation format. All in-text citations now follow a consistent numbered format, and the reference list (pp. 22-23) has been reorganized accordingly to ensure complete consistency throughout the manuscript.

I thank the reviewer for these meticulous suggestions, which have significantly improved the precision and readability of our manuscript. All suggested corrections have been implemented in the revised version. In addition to the changes requested by the reviewer, we have also made further enhancements to strengthen the manuscript, including substantial restructuring of Sections 2 and 3, a revised title, refined abstract and keywords, and an updated author affiliation. These improvements are detailed in the cover letter.

Sincerely,

Zhaorong He (Corresponding Author)
Department of Mathematics, Dongguan University of Technology
Dongguan 523808, China
Email: 17zrhe@stu.edu.cn

February 27, 2026
